# A Narrative Review on Current Diagnostic Imaging Tools for Dentomaxillofacial Abnormalities in Children

**DOI:** 10.3390/children9050621

**Published:** 2022-04-27

**Authors:** Sami Aldhuwayhi, Atul Bhardwaj, Yahya Ahmed M. Deeban, Smita Singh Bhardwaj, Rawan Bakr Alammari, Ayoub Alzunaydi

**Affiliations:** 1 Department of Prosthodontics, College of Dentistry, Majmaah University, Al Majmaah 11952, Saudi Arabia; s.aladdowihi@mu.edu.sa (S.A.); y.deeban@mu.edu.sa (Y.A.M.D.); r.alammari@mu.edu.sa (R.B.A.); 2 Pediatric Dentistry, Department of Preventive Dental Sciences, College of Dentistry, Majmaah University, Al Majmaah 11952, Saudi Arabia; s.bhardwaj@mu.edu.sa; 3 General Dentist, Ministry of Health, Al Artawiyah General Hospital, Al Artawiyah 15719, Saudi Arabia; ayoubauz@gmail.com

**Keywords:** dentomaxillofacial, cone beam CT (CBCT), magnetic resonance imaging (MRI), PET (positron emission tomography), multi-detector row CT (MDCT), ultrasonography (USG), temporomandibular joint (TMJ), 18F-FDG PET/CT (positron emission tomography with 2-deoxy-2-[fluorine-18] fluoro-D-glucose, integrated with computed tomography)

## Abstract

The current review narrates the findings and discusses the available diagnostic tools for detecting structural abnormalities. The review discusses several diagnostic tools, such as magnetic resonance imaging, cone beam computed tomography, multi detector row CT and positron emission tomography. The vital findings and comparative analysis of different diagnostic tools are presented in this review. The present review also discusses the advent of newer technologies, such as the HyperionX9 scanner with less field of view and 18F-FDG PET/CT (positron emission tomography with 2-deoxy-2-[fluorine-18] fluoro-D-glucose, integrated with computed tomography), which can give more efficient imaging of dentomaxillofacial structures. The discussion of effective comparative points enables this review to reveal the available diagnostic tools that can be used in the detection of dentomaxillofacial abnormalities in the pediatric population. The advantages and disadvantages of each tool are discussed, and the findings of past publications are also presented. Overall, this review discusses the technical details and provides a comparative analysis of updated diagnostic techniques for dentomaxillofacial diagnosis.

## 1. Introduction

Any structural abnormality in the dentomaxillofacial region that is congenitally present or is acquired later on can be considered a dentomaxillofacial abnormality. For proper management of any such abnormality, imaging tools are essential as part of the whole diagnostic process. There are many types of oral cavity disorders that may involve the lips, maxillary bones, palate, floor of the mouth and tongue. Many of them can be caused by faulty embryo development or abnormal intrauterine incidence, hampering normal growth of the fetus. These dental malformations may be genetically or environmentally induced (may happen either during morpho- or histo-differentiation time, while teeth are being developed) [1,2]. Despite being asymptomatic, these incongruities bring along with them a host of clinical complications, for example, a delay in or a lack of normal onset of the eruption of teeth or attrition. The infant remains undernourished, due to problems while breast feeding, and may have an unaesthetic dentition, accidental fracture of the cusp or occlusal interference. Disturbance in the tongue space may lead to difficulty in speech and mastication, and pain in the temporo-mandibular joint. Third molars are usually the last teeth to develop, and they may present pathologic disruptions, as compared to other teeth, if they cannot erupt properly. Impacted third molars bring along with them a series of inflammatory and infectious conditions, and the occurrence of cysts and tumors. Furthermore, their extraction is a common practice, which has multiple associated complications. Other perils include malocclusion and disproportionate occlusal force, leading to periodontal problems, tooth breakdown and chances of caries enhancement [3,4,5,6].

Various studies have reported numerous abnormalities, such as “microdontia, talon cusps, congenitally missing teeth, supernumerary teeth, peg-shaped lateral incisors, fusion, gemination, and non-carious defects of enamel” [7,8,9,10]. However, in primary dentition, hyperdontia is uncommon [11]. Brook [10] discovered “0.5% microdontia, 1.6% gemination, and 0.1% dens invaginatus cases” in children in Slough, England. An interesting racial distinction has been noted in cases of dental anomalies, where Caucasians show 0.2% to 1.8%, as compared with 7.8% for Mongoloids [12,13]. Another comparative analysis exhibited cases of hypodontia with a frequency of 0.4% in Swedish children, which lies somewhere in the midway when compared with the 0.0% to 0.9% Caucasian range [12]. For a long time, it has been known and proven that, if a proper dental care regime is followed religiously, all major types of dental caries can be significantly prevented and, to a large extent, moderated by healthy habits [14]. One major preventative behavior is the regular use of a good-quality toothbrush with fluoridated toothpaste and strictly restricted daily sugar consumption [15,16,17,18]. These preventive methods may not always give the desired result, as it depends on the individual who is implementing this sort of healthy behavior [19]. Thus, the dental well-being of children becomes a part of parenting duty, as children are largely dependent on their parents. Thus, involving parents in child oral health programs can help to achieve the desired outcomes [20,21].

## 2. Search Strategy

**Figure 1:**
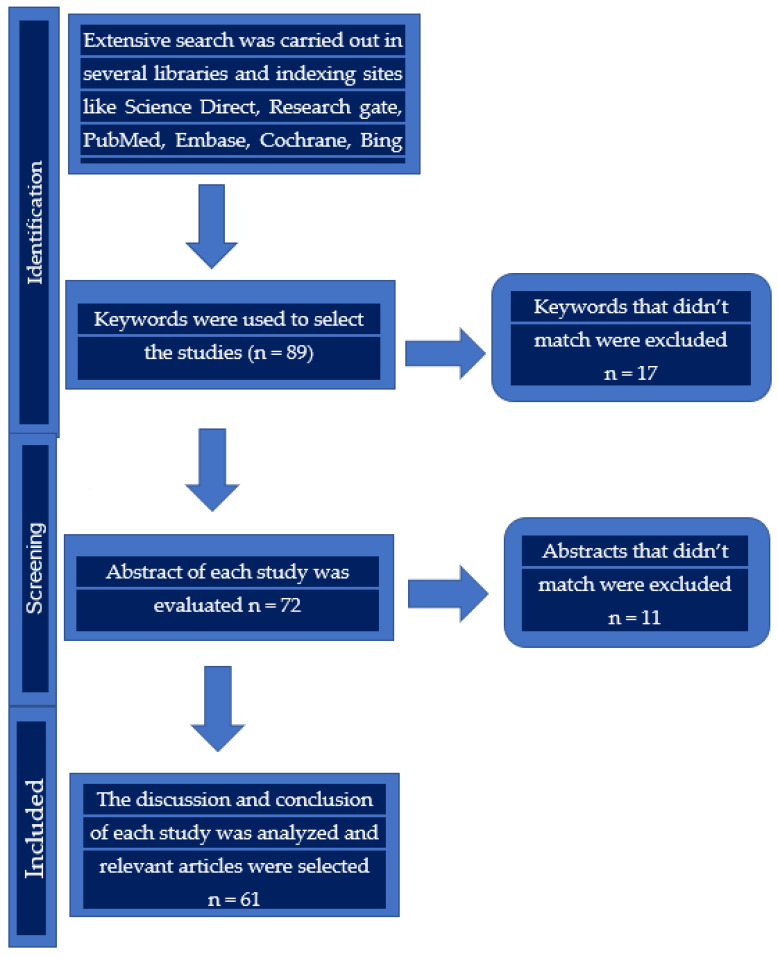
The flow chart of article selection process for this review.

A literature search was carried out in several libraries and on various indexing sites, such as Science Direct, Research gate, PubMed, Embase, Cochrane, Bing Academic, Google Scholar, etc. We also searched the websites of several journals. We mainly searched with keywords, such as (dentomaxillofacial or dental or maxillofacial) and (imaging or imaging diagnostic or diagnosis), (children or pediatric). Several keywords were replaced and tried in different ways to obtain many articles for writing this review. Figure 1 shows the article selection process.

## 3. Epidemiology

According to a rough estimate, approximately 7.8% of people around the globe suffer from major dental problems, including 573 million children [22]. This issue is particularly pronounced among slum populations [23] and in the people of under-developed countries [24]. Dental problems bring with them many negative and personality-related defects, both immediate and long lasting. They can cause pain, difficulty eating, and sleep disruptions, all of which can seriously impede physical development [25,26,27] and oral well-being [28,29]. If an emergency arises and any kind of dental treatment has to be performed with the help of local anesthesia, it poses excessive psychological stress and immense financial losses to the bearer [30,31]. Annual dental treatments cost the NHS (National Health Service) GBP 3.4 billion in the United Kingdom alone [32]. It is interesting to note that dental anomalies can be population dependent, as observed in Nigeria, where the significant difference between the hard dental tissue profile of the native populace and that of Caucasians has been investigated [33,34]. A study by Adeniji revealed that the most common dental anomaly in school-going children was caused by defective enamel (10.4%), 6.7% of which was chronological enamel hypoplasia. The occurrence of hypodontia in two types of dentition was 0.4% (permanent) and 0.05% (primary dentition) [35].

## 4. Diagnostic Imaging Tools

There are several imaging tools, but only those that are used in imaging for dentomaxillofacial abnormalities in children, whether acquired or congenital, are discussed here.

**Figure 2:**
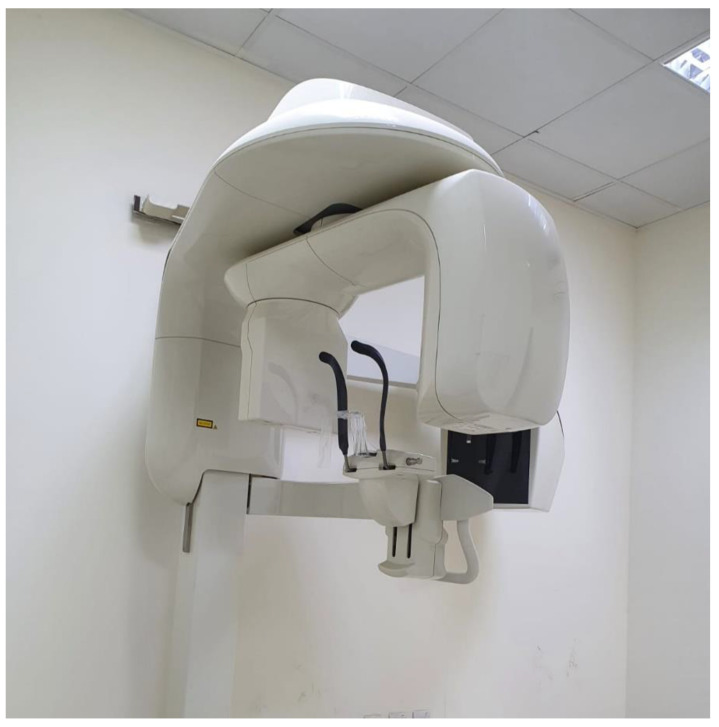
CBCT machine.

Cone beam CT (CBCT) (Figure 2) is commonly considered as the first line of imaging, and it utilizes a “pulsed conic or pyramidal beam of X-ray with a flat panel detector” [36]. A one-time rotation in a round path takes place around an isocenter, and then the images are obtained [36]. The level of radiation exposure that is required in CBCT is less than in MDCT (multi-detector row CT) [37]. Small sets of images that vary from 4 cm (ideal for a few teeth) to more than 20 cm in diameter are easily provided by CBCT. According to the clinical indication [34,35,36,37,38], there are many patients who are claustrophobic, and in their case, CBCT is the best option, as it has an open-ended design and can be executed in two upright positions (both standing and sitting) [36]. Its main drawback is that, while targeting the soft tissues, the image quality is not up to the mark and may produce metal artifacts [36]. This method facilitates multiplanar reformatting and 3D reconstruction [39]. Authors have documented that CBCT can be used in surgical, endodontic, implant and orthodontic cases of the dentoalveolar region, and for diagnosing maxillofacial pathology [40].

MDCT has a fan-shaped beam and images are obtained by a number of rotations performed around the patient. They rotate “in a spiral motion over axial plane” [41]. It has a shorter acquisition time, which considerably reduces motion artifacts (which might happen due to breathing and swallowing). The most prominent feature of this tool is that, while imaging the soft tissue, it renders superior characterization. This method allows the usage of a contrast agent, which is iodine-based and can be of great help when considering an infection or tumor in the dentomaxillofacial region [41]. This method has three major setbacks: it is not cost effective; the machinery needs a large amount of space, as it is, physically, a space-occupying machine; and this method produces metal artifacts [42]. In this case, tailored, separately-purchased dental software packages deliver “multiple cross-sectional, panoramic images along dental arches,” which form an essential part of the dental implant strategy [43]. While performing this imaging, the use of the “puffed-cheek” method ensures that a detailed image with improved accuracy will be obtained [44]. However, when compared with one another, dental CBCT has been found to be better than MDCT in both dental and implant modes [45]. Authors have documented that the image quality of CBCT is better than MDCT. However, the contrast resolution is lower than MDCT. Hence, MDCT can be used for the imaging of soft tissues, while CBCT can be used for the imaging of maxillofacial hard tissues [46].

Magnetic resonance imaging (MRI) is one of the most frequently used tools that is free of radiation exposure, which has made it effective for detecting dentomaxillofacial lesions in children. In cases of selected dentoalveolar diseases, it efficiently provides a high-quality contrast of soft tissue. Magnetic resonance imaging is the ideal imaging investigation for scanning the head, spine and joints, osteomyelitis cases, infections of soft tissues and jaw tumors [47,48,49]. It can also analyze the terminal branches of the trigeminal nerve, which lie near to the mandibular third molars [49]. It has two disadvantages: it is expensive and its spatial resolution is lower than CT (computed tomography) [50]. Previously, authors have recommended that patients suffering from temporomandibular joint symptoms should undergo assessment by MRI to ease the selection of the appropriate therapy for them [51]. Authors have documented that MRI clearly performs the imaging of malformed teeth, pulp, pulp canals, and the cortical bone in three dimensions, is useful for performing orthodontic and surgical treatment in children, and can be used for repeated imaging [52].

There has been a modification in the existing diagnostic tools. A CBCT image can be captured using an optical revenge dental device (HyperionX9/Open Tech 3D Srl) scanner, which has a field of view (FoV) of 5 cm × 11 cm, keeping the patient’s exposure to a minimum level. The captured image can be scanned by the Optical Revenge Dental device, and then converted into an STL (stereolithography) extension using software. This allows clinicians and technologists to better formulate or design functional devices, such as implants for dentomaxillofacial abnormalities. Hence, the diagnostic tools that are already available can also be employed to enhance their applicability to design a solution by which the patient can cope with the existing structural abnormality. As all these techniques are virtually operated, radiation exposure is kept at the minimum possible level. These virtual techniques are effectively adapted for children, helpful in the 3D assessment of endodontic lesions, and, thereby, should be further enhanced for diagnostics [49,50,53].

Several studies have explored the possibilities of nuclear medicine diagnosing lesions in the maxillofacial region. Diagnostic nuclear medicine deals with the 99mTc-MDP, which has been used for the detection of skeletal metastasis in prostate and bladder carcinoma cases. The same techniques can be implemented in detecting lesions in the temporomandibular joint, cases of condylar hyperplasia, Paget’s disease, and bone grafts. Positron emission tomography (PET) with 2-deoxy-2-[fluorine-18] fluoro-D-glucose, combined with computed tomography (18F-FDG PET/CT), is more clinically effective than any other tool available, because the structural abnormality can be viewed from multiple angles. The efficient 3D structuring using software and the efficiency of diagnosing minute details of the dentomaxillofacial region are the reasons behind its increasing usage. Due to its higher cost though, there is a limit to its usage [54]. However, if PET along with CT can be used, this diagnostic approach can change the therapeutic approach, due to its high sensitivity (89%) and specificity (95%). A PET diagnostic tool can be used in combination with CT or MRI, depending upon the target lesions or abnormalities [55].

**Figure 3:**
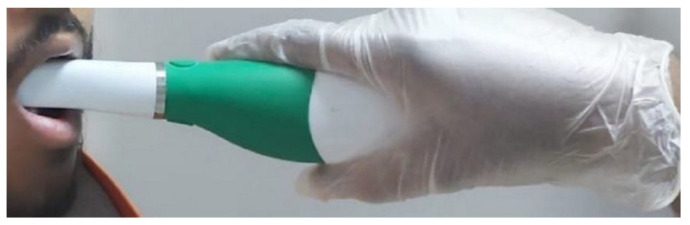
Intra-oral ultrasonography.

Although CBCT was traditionally chosen as the first line of investigation for dentomaxillofacial abnormalities, the usage of ultrasonography (USG) (Figure 3) in the investigation of the dentomaxillofacial region is gaining momentum as the first line of investigation. This is due to the increasing radiation exposure that occurs from CT investigation. Due to the absence of radiation exposure, ultrasound is one of the best investigative tools in the pediatric population. Ultrasound is efficient in visualizing finer details of the surface tissue structure in the dentomaxillofacial region, without any ionizing radiation exposure. The drawbacks that are documented for this tool are less spatial resolution and reduced penetration into structures filled with gases or bony structures. Conversely, the advantages are that ultrasound is clinically efficient in determining the thickness of muscles, studying the vessels and soft tissues of the neck region and TMJ (temporomandibular joint), and visualizing vascular lesions and lymph node abnormalities. The evaluation of periapical lesions can best be performed by ultrasound. Finally, ultrasound is one of the most economical tools available today [56,57,58]. A study conducted in vitro compared conventional radiographs with CT, MRI and USG for the detection of foreign bodies in soft tissues, and observed USG to be very efficient, in terms of sensitivity and specificity [59]. Another study used USG for monitoring the healing of periapical tissues post-surgery, and found that USG gave better results than conventional radiographs [60].

## 5. Rationale

This narrative review has discussed the investigative tools that are being used today in the evaluation of dentomaxillofacial abnormalities, in particular, in the pediatric population. It has been mentioned that CBCT is considered the first-line tool traditionally, but due to the absence of radiation exposure and cost effectiveness, USG has gained popularity as the first line of investigation. Finally, it depends on the clinician to prescribe investigation according to their suspicion of the condition present in a given case [1,2,3,4,5,6,7,8,9,10,55,56]. The following Table 1 summarizes the investigative tools, with respect to the conditions that can be best diagnosed with these tools.

The cost of these investigative tools varies, and is different in all countries. In addition, many factors, such as the country’s tax, health expenditure index, local government regulations, etc., come into play when the prices are decided. Roughly, it can be observed that ultrasound is the most cost effective, followed by CBCT. While, for better results and advanced cases, MRI is preferred. For life-threatening conditions, PET scans can be prescribed. As a first-line investigation, USG may be preferred in cases where soft tissue and lymph node abnormalities are suspected; while, in cases of maxillofacial trauma, and jaw and facial bone abnormalities, CBCT can be prescribed [1,2,3,4,5,6,7,8,9,10,42,43,53,54].

Regarding the radiation dose, dentists should never solely think about earning money and expose children to unwanted radiation. Three principles should be followed: (1) “Justification principle” (radiographs should only be taken when there is no other way of obtaining relevant information. If the patient is not able to cooperate, then it should not be performed). (2) “Limitation principle”; dentists should always follow the ALADAIP principle (as low as diagnostically acceptable, being indication oriented and patient specific). Extremely low doses may produce images that are not diagnostically useful. However, we need diagnostically acceptable images for each specific indication. The reference levels for radiation doses should be compared with either regional, national or international levels that will indicate the approximate dose levels for various medical procedures. Unnecessary and repeated examinations must be avoided, with some exceptional use as in the case of cancer treatment. (3) “Optimization principle” (dentists should always try to obtain the radiographic image using the above two principles). A reduction in dose for CBCT can be achieved by a reduction in the FOV (field of view) [61,62]. MRI is a radiation-free tool that can assess the terminal branches of nerves and minute details. USG has several advantages, such as low cost and the absence of radiation exposure.

Dentomaxillofacial diagnosis is mostly performed by CBCT. This review has highlighted less commonly used tools, such as PET/CT or PET/MRI. The current review has summarized each diagnostic tool for various dentomaxillofacial conditions. It has presented various concepts, based on which clinical prescription can be performed, based on investigations for the efficient evaluation of dentomaxillofacial conditions, especially in the pediatric population. This also involves considering the socio-economic parameters of the patient. This review has discussed every detail of the tools that can be used in pediatric imaging for diagnosing abnormalities in the dentomaxillofacial region. In practice, there are many factors that come into play when choosing the right diagnostic tool.

## 6. Conclusions

PET enhances the possibility of detection of abnormalities significantly. The advancement of the use of PET with CT should lead to a reduction in radiation exposure and allow for extensive usage in the pediatric population. In cases of dentomaxillofacial tumor or infection, MDCT is an effective tool, but poor cost effectiveness has led to its limited use. MRI and USG do not use radioactivity. MRI is comparatively expensive, whereas USG is cost effective.

## Figures and Tables

**Table 1:** Investigative tools for the diagnosis of various dentomaxillofacial abnormalities.

Investigative Tool	Condition Likely to Be Diagnosed Effectively
Cone beam CT (CBCT)	Planning of dental implants, visualization of abnormal teeth, evaluation of jaws and face, assessment of cleft palate, dental caries diagnosis, endodontic diagnosis, and diagnosis of dentomaxillofacial trauma
Multi-detector row CT (MDCT)	Soft tissue characterization
Magnetic resonance imaging (MRI)	The high-grade contrast of soft tissue, dentoalveolar diseases, soft tissue infections, cystic and solid components of dentomaxillofacial tumors, to assess trigeminal nerve terminals
Positron emission tomography (PET)	Efficient 3D structuring in the temporomandibular joint, condylar hyperplasia, Paget’s disease and bone graft cases
Ultrasonography (USG)	Thickness of muscles and vessels of the neck region, visualization of vascular lesions and lymph node abnormalities, evaluation of periapical lesions

## Data Availability

Not applicable.
